# A Narrative Review of Soccer-Related Concussion Management in Children and Adults Over the Past 10 Years

**DOI:** 10.7759/cureus.67510

**Published:** 2024-08-22

**Authors:** Pamela Castillo Rocha, Maria D Beletanga, Osvaldo Pangrazio, Francisco Forriol, Christopher Howards, Mildred C Franco-Liñan, Gabriela Restrepo-Rodas, Daniela P Benitez Gutierrez, Andrea Perez, Jeffrey Neuman, Alcy R Torres

**Affiliations:** 1 Neurology, Nicklaus Children's Hospital, Miami, USA; 2 Boston Medical Center, Boston University Chobanian & Avedisian School of Medicine, Boston, USA; 3 Orthopedics, CONMEBOL (Confederación Sudamericana de Fútbol) South American Football Confederation, Luque, PRY; 4 Antidoping, CONMEBOL (Confederación Sudamericana de Fútbol) South American Football Confederation, Luque, PRY; 5 Management, WUSC (Wellesley United Soccer Club), Wellesley, USA; 6 Neurology, Boston University Chobanian & Avedisian School of Medicine, Boston, USA; 7 Neurology, Nova Southeastern University, Fort Lauderdale, USA; 8 Pediatrics, Boston University Chobanian & Avedisian School of Medicine, Boston, USA

**Keywords:** management, mechanism of injury, youth soccer, concussion, traumatic brain injury

## Abstract

Soccer-related concussions (SRC) have increased despite an overall reduction of concussions across all sports activities. Few papers have studied the mechanism of injury, and have been mostly done in high-income countries or focused on small populations, preventing generalization. Our goal was to analyze the available data published about SRC over the past 10 years, independent of the country's income level.

A narrative review was performed. The definition of sport-related concussion from the American Academy of Neurology and studies published between 2013 and 2023 were used. Of 1210 articles, 45 met the inclusion criteria.

The results showed that SRC was more frequent in females (57.6%) than males (44.3%). Player-to-player interaction was the most common mechanism of injury, with midfielders being the most affected position. The first providers to diagnose were certified athletic trainers, within the first 24 hours. Neurological evaluations, including SCAT (Sport Assessment Concussion Tool) and ImPact (Immediate Post-concussion Assessment and Cognitive Testing), were included in 42.2% of the studies, with SCAT and ImPact specifically used in 15.5% and 11% of cases, respectively. Need for hospitalization was found in 8.9% of participants and one player required surgical intervention. At the time of the concussion, confusion, dizziness, and amnesia were reported frequently. However, after the concussion, headaches and dizziness were prevalent. Follow-up data were included in 35.5% of the studies. On average, children missed 15 practice days and returned to school after 8 days.

In conclusion, future research should focus on the circumstances around head-to-head injuries by age, sex, and level of professionalism as well as the importance of early diagnosis and careful follow-up, to protect the players and improve their outcomes.

## Introduction and background

Soccer is one of the most popular and practiced sports worldwide “with over 265 million active players worldwide”[1]. An estimated 283,000 children seek care in U.S. emergency departments each year for a sport- or recreation-related traumatic brain injury (TBI). TBIs sustained in contact sports account for approximately 45% of these visits. Football, cycling, basketball, playground activities, and soccer account for the highest number of emergency department visits [2].

With soccer gaining popularity, soccer-related concussions have increased despite the overall reduction in the number of concussions across all sports activities in both pediatric and adult populations [1]. The U.S. has the largest number of publications [3]. Concussion research in high-income countries is still relatively young, but it has been able to ascertain that concussions are not trivial injuries and can potentially lead to long-term neurological sequelae, especially in those with multiple occurrences. In the past decade alone, we have seen changes in several sports, at all levels, to help prevent and better manage concussion injuries. Soccer is a global sport, with more participants of all ages than any other sport [4].

Certified athletic trainers are the first providers to diagnose soccer-related concussions in high-income countries, raising concern for its diagnosis in low-income countries. We must keep in mind that health disparities are prevalent in public health around the globe, likely affecting patient outcomes. In addition to its vast number of participants, soccer is also popular in low and middle-income countries that lack the means for effective education and management of concussions. The majority of current concussion research is conducted in high-income countries (HICs), creating a disparity in knowledge of concussion diagnosis and treatment [4].

The present review aims to gain a better understanding of the mechanism of concussions and to underscore the importance of refining research methodologies, standardizing assessment protocols, and directing attention to specific player demographics to advance our understanding of soccer-related concussions and enhance player welfare, aiding healthcare providers in more effective recognition, management, and prevention.

## Review

Materials and methods

According to the American Association of Neurological Surgeons, a concussion is a brain injury resulting in temporary loss of normal function. This can include an alteration in mental status or level of consciousness resulting from a kinetic or mechanical trauma to the head [5].

Based on the above definition and the Participants, Intervention, Comparison, Outcome, and Study design (PICOS) format, our research questions were: what are the predominant mechanisms leading to soccer injuries, the prevalent neurological findings associated with reports in standardized evaluations, and the field positions most frequently implicated in concussions, considering both pediatric and adult populations in the last 10 years? The research question was formulated as follows: (P): pediatric populations (up to 21 years old) versus adults (above 21 years old); (I): diagnosis of soccer-related concussion based on the definition of Sport-Related Concussion from the American Academy of Neurology or by author criteria; (C): characteristics and mechanisms of concussion; (O): Field positions most frequently involved in soccer-related concussions, the prevalence of neurologic symptoms reported, and the prevalence of soccer-related concussion studies that used standardized neurological evaluations for diagnosis over the specified period; (S): primary studies. The search strategy is as follows: (((Concussion) OR (Head trauma) OR (Cerebral concussion*) OR ("Brain concussion" [Mesh])) AND ((Football) OR (Soccer)) AND ((Pediatric*) OR (Child*) OR ("Pediatrics"[Mesh]) OR ("Adult"[Mesh]) OR (Adult))). 

Inclusion criteria comprised the following: soccer-related concussion causes, mechanisms, type of injury, and consequences of the impact in males and females of all ages. Primary studies (clinical trials, case-control, case series, case reports, cohort studies) in English and Spanish published in the last 10 years, from 2013 to 2023, in PubMed were considered and filtered by the platform Rayyan. The search for individual studies was done by manual searches of reference lists on systematic reviews. Secondary studies and others done before 2013 were excluded. Four authors carried out the selection of studies and meetings were planned to discuss the studies for which a divergent selection decision was made.

We found 1210 published studies in our database search. After the removal of duplicates, we screened 1194 manuscripts based on title and abstracts, ending up with 120 papers after the first filter. Full-text documents were read and only 45 met our inclusion criteria. We searched documents that cited any of the initially included studies as well as the references of the initially included studies. However, no extra articles that fulfilled the inclusion criteria were found in these searches. We tried to contact some study authors, but because of time constraints and no answers, we could not clarify some information. However, we are certain that none of these methodological limitations would change the overall conclusions.

We excluded 1074 studies because most of them had head concussions while playing American football or other contact sports and not exclusively soccer-related concussions. Some of them did not meet the criteria of concussion. Additionally, some of them did not report the method of diagnosing the concussion and did not use “concussion” according to its definition by the American Academy of Neurology.

The data collected from the various publications were organized and tracked in an Excel file (Microsoft Corporation, Redmond, WA, US). After validating the data, they were exported into a Python script, which was used to process and visualize the results. The packages Matplotlib and Seaborn were used to make the figures. This methodology ensured a cohesive and efficient workflow from initial data handling to the final visual representation of the figures. Basic mathematics was used. The Python script, with both the data processing and visualization code, is readily available to reproduce any of the figures.

Equity, diversity, and inclusion statement

In our data collection process, we ensured that all studies were included regardless of gender, religion, demographics, and language. Our team of researchers comes from diverse backgrounds, which helps minimize the exclusion of studies. All results were evaluated with equity, irrespective of the authors’ backgrounds, the number of citations, or the journals in which the articles were published. This method of stratification allowed us to discuss the overall generalizability of our results.

Results

**Figure 1:**
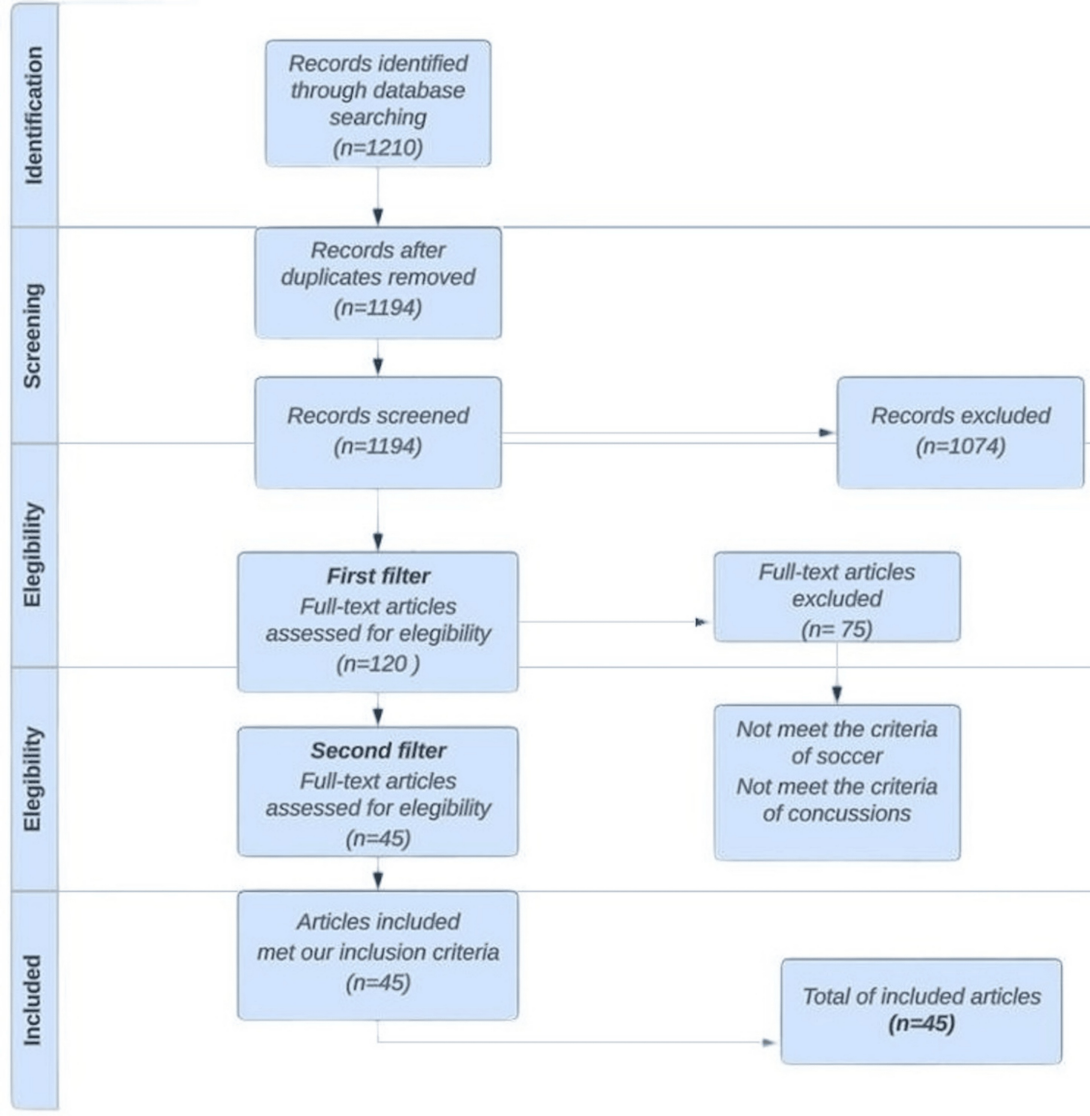
Flowchart inclusion and exclusion studies

After reviewing literature focused on soccer-related concussions, our review of 45 articles meeting the established inclusion criteria unveiled key insights into the global approaches to concussion management (Figure 1).

**Figure 2:**
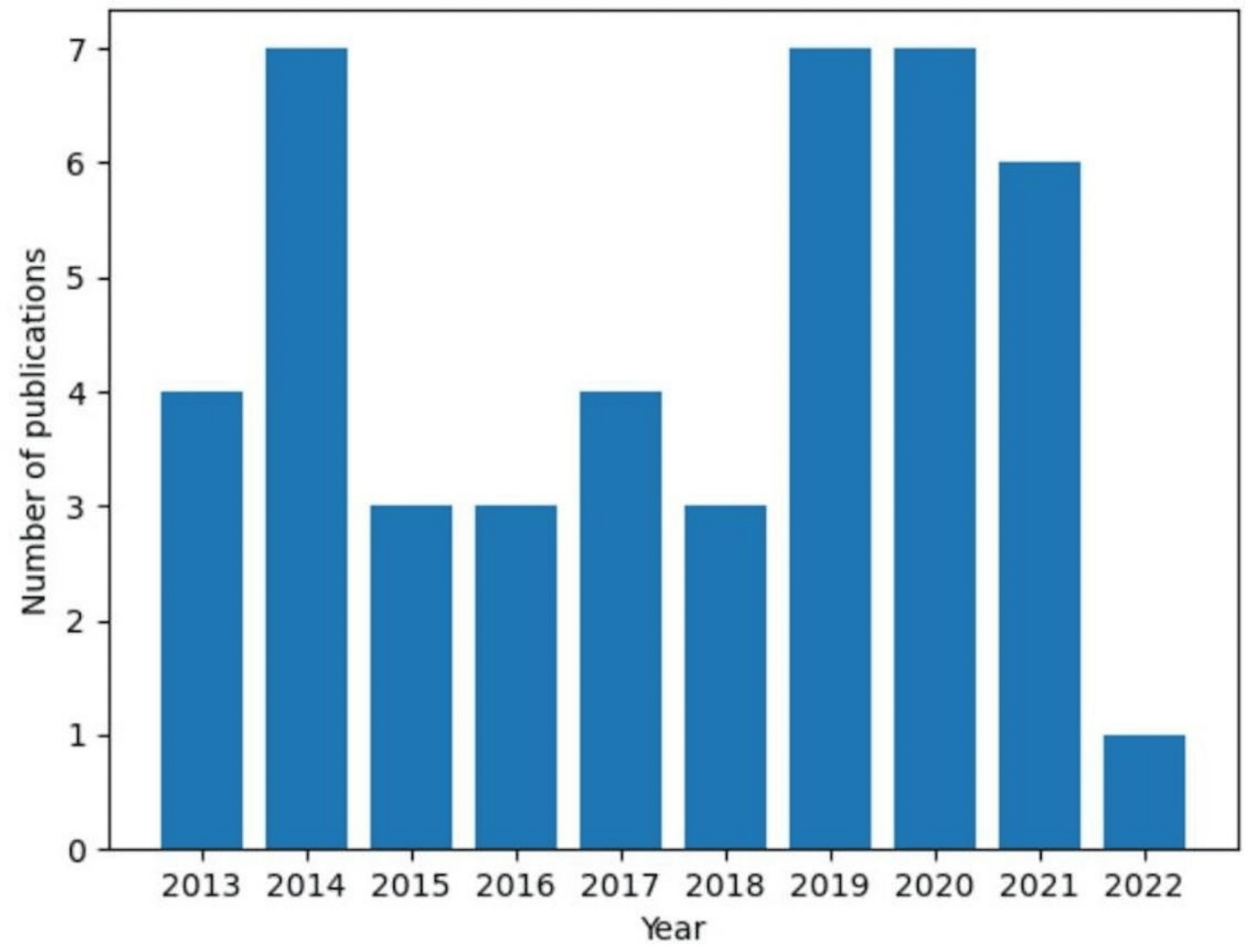
Concussion research publications from 2013 to 2022 Axes: The x-axis displays the years between 2013 and 2022 while the y-axis represents the number of publications on concussions. Over the span of 2013 to 2022, there has been an average of 4.5 publications per year on the topic of concussions, with a standard deviation of 2.12. Interestingly, the number of publications experienced a decline, reaching just 1 in the year 2022. On the other hand, the years 2024, 2019, and 2020 stand out as the peak periods for research on concussions, exhibiting an average of 7 publications during those years.

Our review found that 42.2% of the studies included neurological evaluation as part of their concussion protocols. From this percentage, 15.5% used the Sport Concussion Assessment Tool (SCAT), a post-concussive symptom questionnaire designed to provide a standardized measure of concussion-related symptoms. The SCAT tools are most effective in discriminating between concussed and non-concussed athletes within 72 hours of injury and up to 7 days post-injury, although their clinical utility appears to diminish after 72 hours [6]. This finding underscores a potential gap in the comprehensive assessment of concussions, with the lack of neurological evaluation and the use of standardized tools in more than three-quarters of the studies analyzed raising questions about the adequacy of these assessments. The trend of increased concussion reporting likely reflects heightened education and awareness following litigation and increased media coverage (Figure 2) [1].

**Figure 3:**
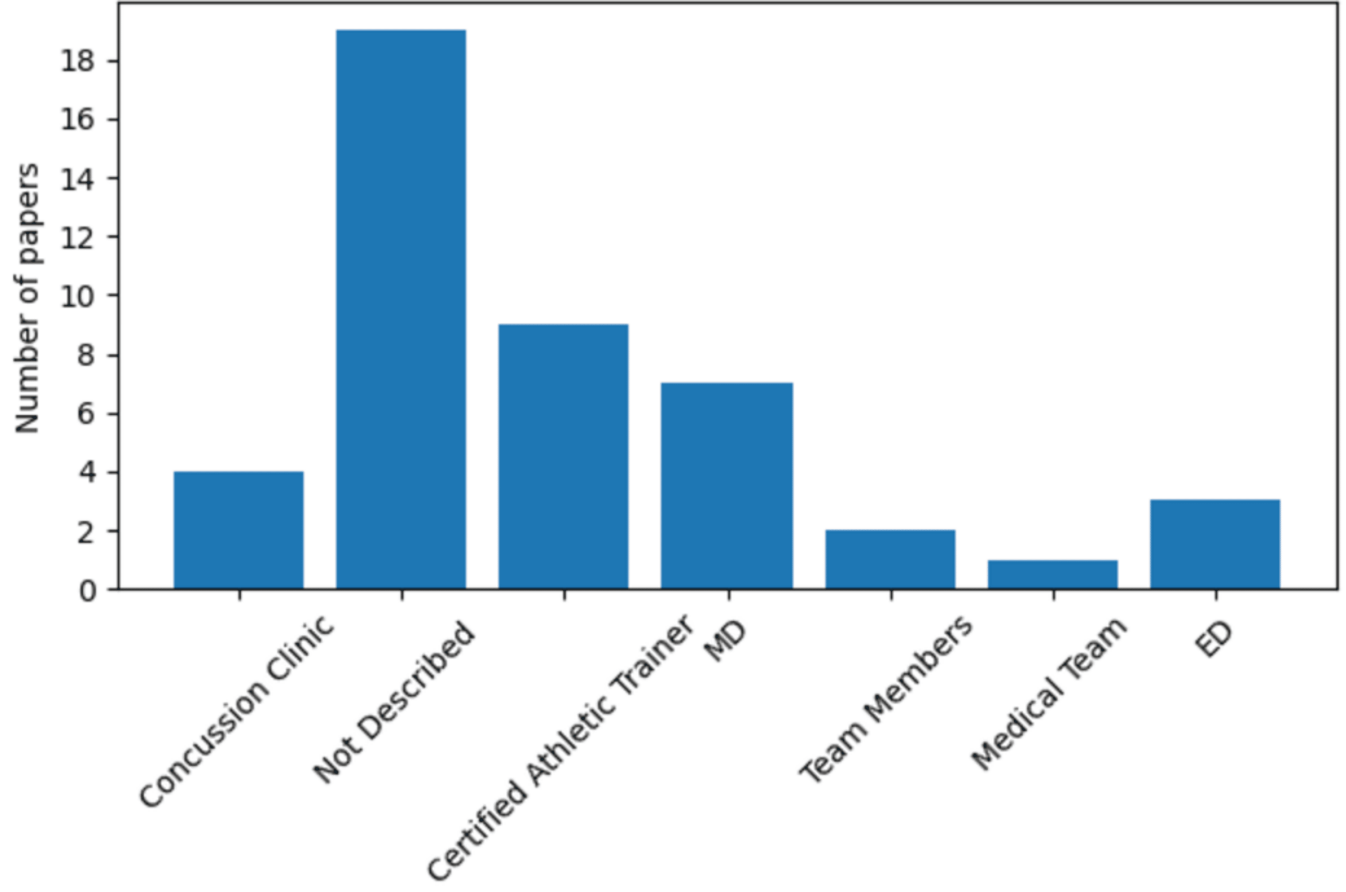
Primary provider diagnosing a concussion An analysis Axes: The x-axis categorizes various scenarios and providers involved in diagnosing concussions, including concussion clinics, certified athletic trainers, medical doctors (MDs), team members, the medical team, emergency physicians, and unspecified providers. The y-axis represents the number of publications mentioning each provider, ranging from 0 to 18. The bar graph provides insights into the most frequent providers mentioned in the diagnosis of concussions based on a collection of publications. Notably, a substantial number of publications, totaling 18, do not specify the provider involved in diagnosing concussions. Following this, certified athletic trainers emerge as the most mentioned primary providers diagnosing a concussion, with nine publications reporting their involvement. MDs also play a significant role, being cited in seven publications as the primary diagnosing providers for concussions.

The inclusion of neurological evaluation is essential for understanding the cognitive implications of concussions and could contribute to the development of more efficient diagnostic and management strategies. These limitations highlight the importance of using robust, multitiered, and age-appropriate normative data in the interpretation of SCAT and other acute measurement tools [6]. It is important to emphasize that in the majority of articles, the athletic trainer was responsible for the evaluation (Figure 3).

Although SCAT tools are designed to be self-sufficient without the need for ancillary equipment [6], prior knowledge about concussions is essential. Having individuals with the necessary professional qualifications to perform the evaluations is crucial.

**Figure 4:**
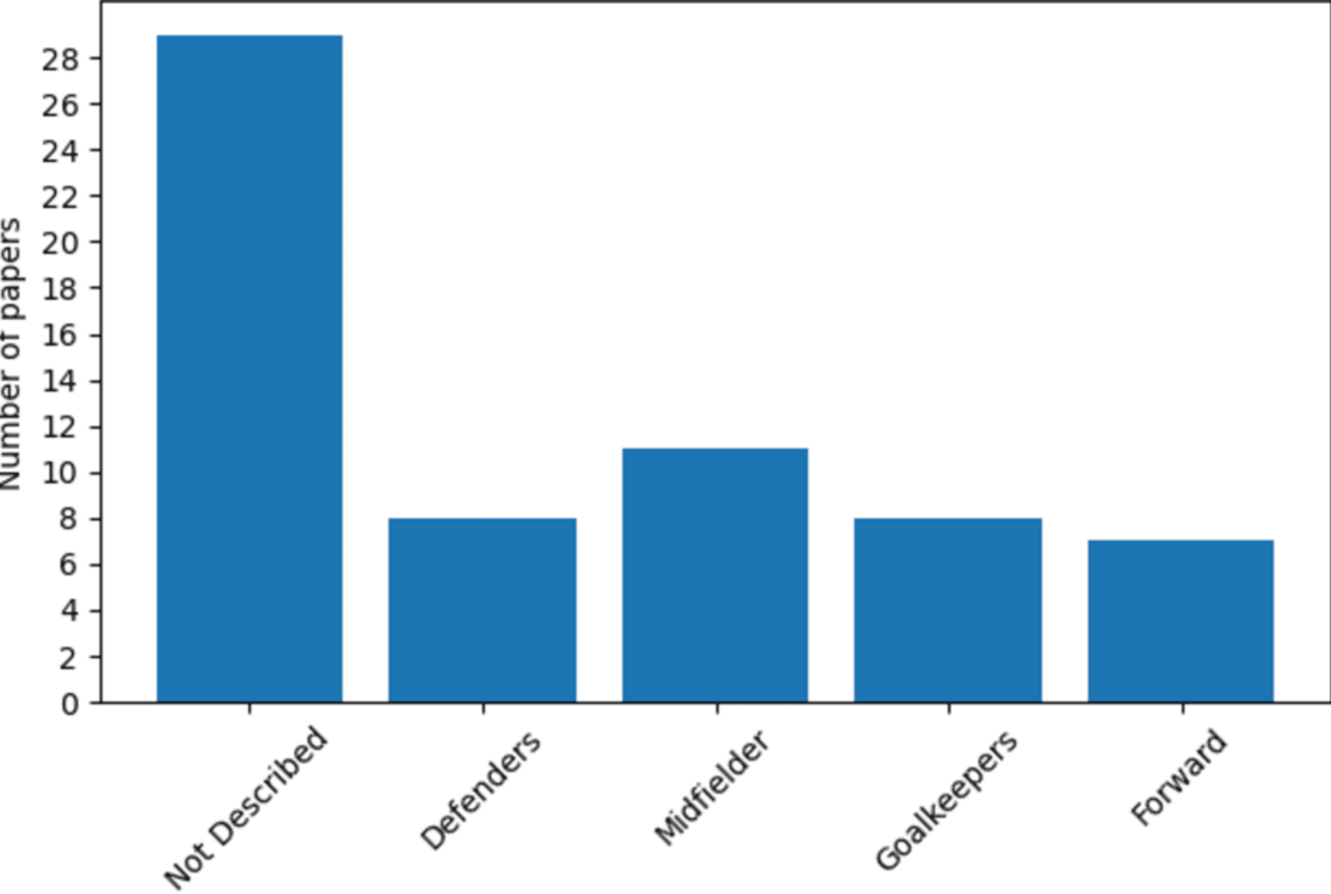
Prevalent field positions linked to concussions An analysis Axes: The x-axis displays various field positions, including defenders, midfielders, goalkeepers, forwards, and positions not described. The y-axis represents the number of papers, ranging from 0 to 28, which mention each field position. The presented bar graph provides insights into the most frequently mentioned field positions associated with concussions in a total of 28 papers. Surprisingly, a significant number of papers, 28 in total, do not specify the exact field position related to the concussions. However, among the papers that do identify positions, midfielders emerge as the most common, with a substantial count of 12 papers. The third most prevalent field positions connected to concussions are defenders and goalkeepers, both mentioned in eight papers each. Lastly, the forward position appears to be the least common, being mentioned in only seven papers.

The analysis exposed that midfielders experience a higher frequency of concussions resulting from player-player interactions (Figure 4).

The most common mechanism of injury, identified as "combined", refers to the interplay between the arm-elbow-head [7]. This observation is consistent with the physically demanding characteristics of the midfield position. Players are responsible for recovering and distributing the ball effectively; they act as a link between the defensive and offensive units of the team. They are positioned in the middle third of the field, frequently involved in controlling and distributing the ball [8]. In fact, it is very well known in soccer that the team that wins the midfield might win the game. Acknowledging these positional susceptibilities is essential for adapting preventative approaches and increasing attentiveness in monitoring midfielders during matches.

Including follow-up data, a critical component for tracking the progression and resolution of concussion symptoms, was observed in 35.55% of the studies. While this represents an important portion of the literature, the absence of follow-up in a significant number of studies underscores the need for a more uniform approach to long-term observation in soccer-related concussion research. The risk of going back to play without a protocol of gradual reinsertion increases the probability of children suffering new concussions that could result in long-term neurologic consequences. Those athletes who suffered at least two concussions took longer to return to play after a second concussion [9]. The integration of extended follow-up periods is essential for understanding the recovery trajectory and potential complications. Results of long-term follow-ups lead to evidence-based return-to-play and return-to-school guidelines.

To protect the athletes, especially children, a return to play law was created in some states. To assess the implementation of return-to-play laws, the National Center for Injury Prevention and Control (NCIPC) established the guidelines. Unfortunately, the majority of concussed players (59.3%) continued to play with symptoms, and there was no statistically significant difference in the proportion of players who played with symptoms before and after the law was passed [10]. Many athletes go back to play within 24 hours after a concussion and many of them still have symptoms.

**Figure 5:**
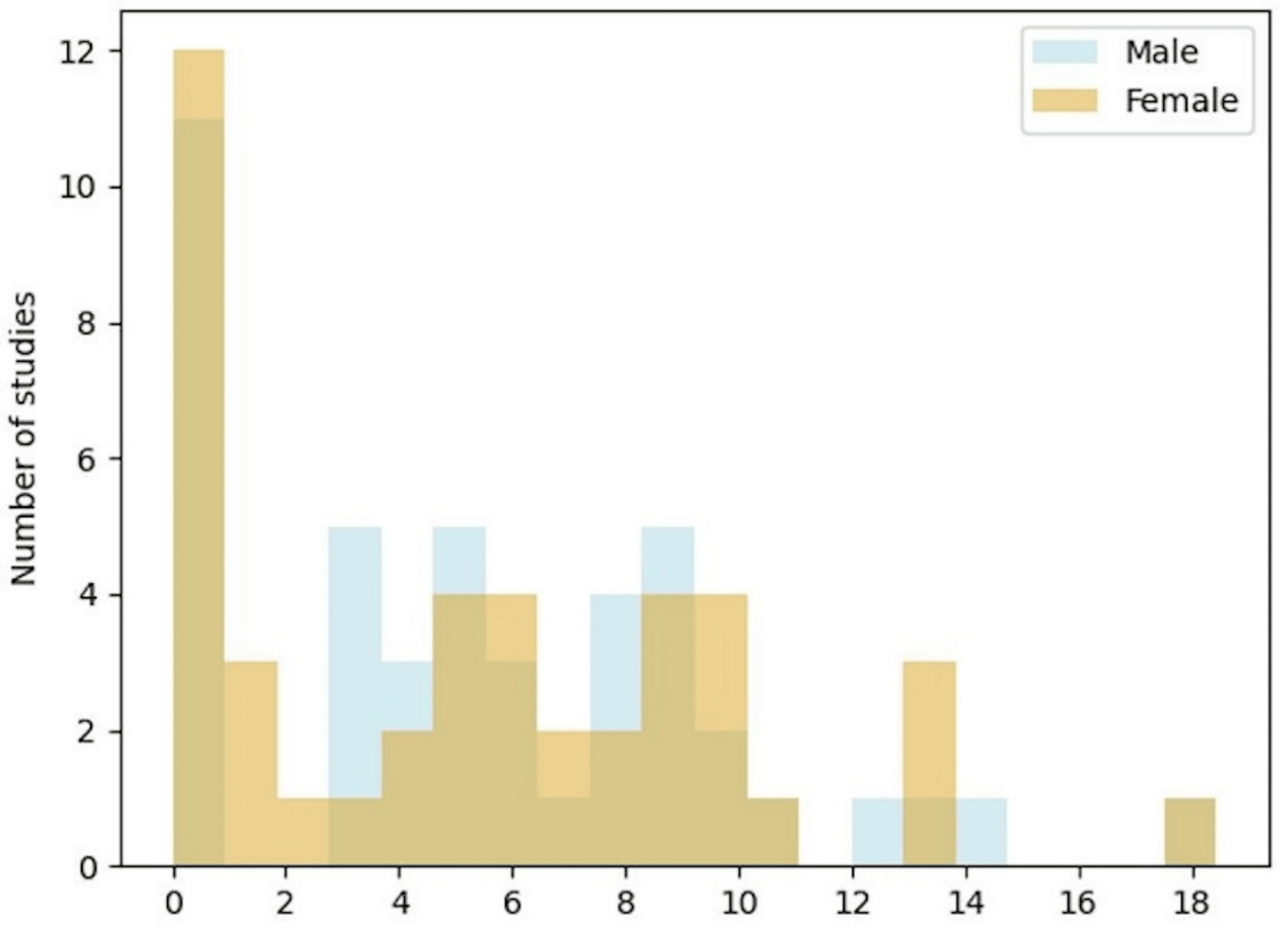
Number of players per study Axes: The x-axis is the log2 scale of the number of players in the study. The y-axis is the number of studies. In the bar graph, it is evident that a significant proportion of studies have a limited number of participants, with over 12 studies having less than 10 people each. Moreover, the graph shows an equal representation of both male and female players across the various studies. Nevertheless, there is one notable exception where a considerably larger study focused on 100,000 more females than males.

The average duration of return to school or activities was identified as eight days across the studies. This measure offers a valuable understanding of the overall recovery timeline and can act as a reference for healthcare professionals, educators, and parents engaged in the recovery and reintegration processes for soccer players who have experienced concussions (Figure 5).

This review aims to spot critical aspects of soccer-related concussions, emphasizing the need for standardized assessment protocols, extended follow-up practices, and position-specific considerations, especially for midfielders. The integration of certified athletic trainers in the evaluation process and insights into the average return to school/activities collectively contribute to advancing our understanding and management of soccer concussions. Addressing these gaps will not only contribute to the improvement of diagnostic and management approaches but also aid in the development of targeted preventive strategies to protect the well-being of soccer players.

Discussion

**Figure 6:**
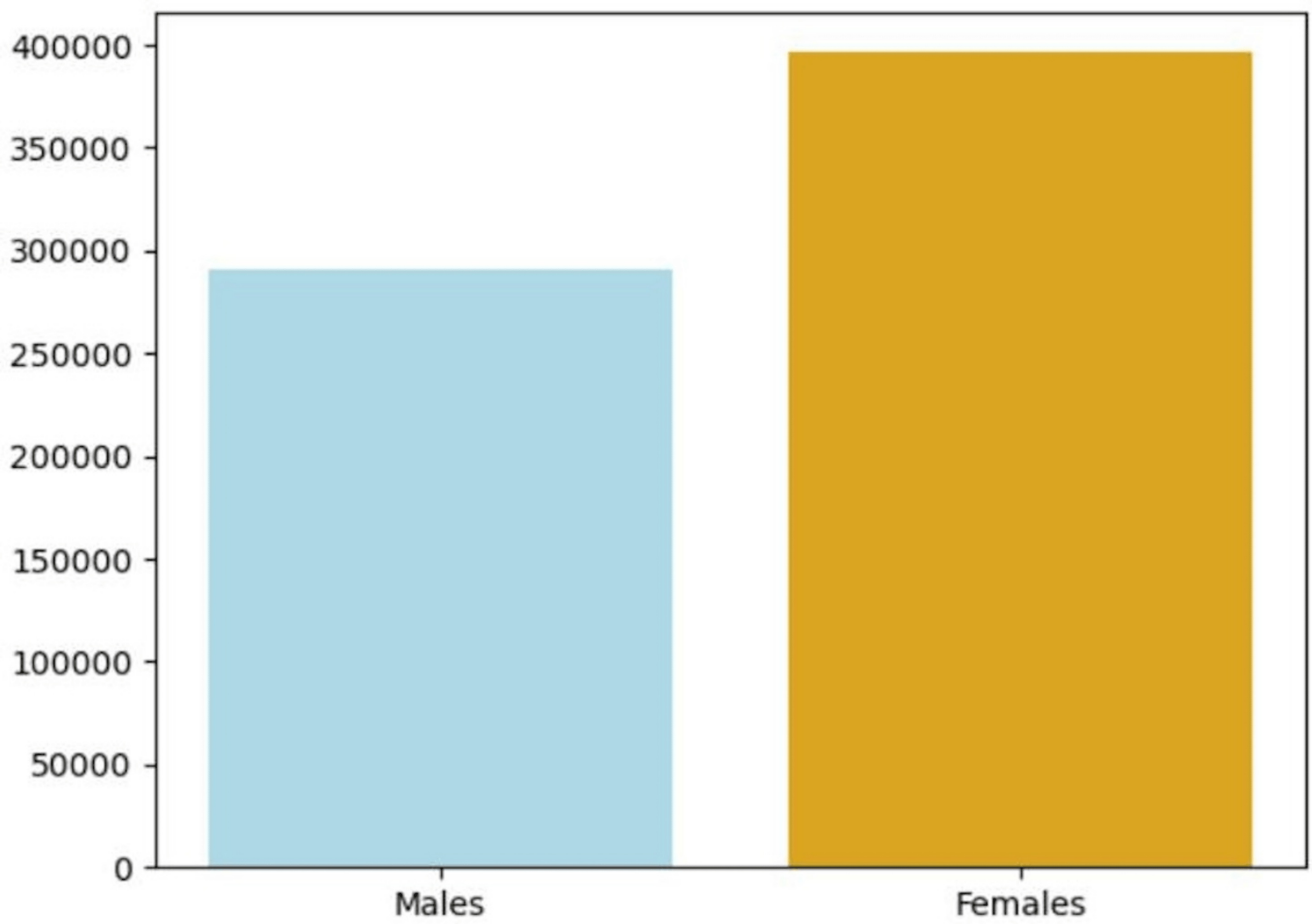
Concussion case reports by gender The presented bar graph highlights gender-specific concussion case reports, revealing a notably higher number of cases reported among females, totaling 394,429 instances. Comparatively, male individuals accounted for a total of 290,876 reported cases associated with concussions.

Analyzing soccer-related injuries reported through online surveillance in U.S. soccer programs unveiled a compelling narrative. Concussions accounted for 20% of injuries during high school soccer competitions and 8% in collegiate soccer competitions. They emerged as one of the most prevalent injuries across all positions during competitive matches [11], with midfielders being the most commonly affected. A retrospective analysis of injuries evaluated in Canadian emergency departments emphasized the demographic disparities in concussion occurrences. The number of concussions was higher among males from 10 to 14 years old and among females from 15 to 19 years old than among other age groups (Figure 6) [12].

These findings highlight the importance of the development of comprehensive strategies to mitigate both the occurrence and the consequences of concussions within this sport.

Symptoms

The studies collected and analyzed during this review offer a broad landscape surrounding soccer-related concussion symptomatology. A descriptive epidemiological study performed by Khodaee et al. unveiled that the most prevalent symptoms experienced by concussed individuals were headaches (92.6%), dizziness/unsteadiness (68.8%), and concentration difficulties (52.2%). Less frequent symptoms included loss of consciousness (3.6%) and amnesia (15.8%). Regarding the mechanism of injury, this study highlighted that the most frequent mechanism of concussion was player-to-player contact during heading [13]. Similarly, a European study performed on professional soccer players found that players are more likely to be injured during official matches than in training sessions, with a concussion rate 78-fold higher in match plays [14].

Reintegration After Injury

The reintegration of athletes into soccer following a concussion commonly leads to disagreements between athletes, coaches, and medical professionals. A retrospective study revealed that player’s attitudes regarding returning to play after a concussion varied depending on a history of previous concussions and the availability of immediate evaluation. Players with a history of prior concussions were more likely not to continue to play when suffering a concussion during a match than those who did not have a previous history. Likewise, soccer players who were evaluated immediately after the injury had greater odds of not continuing to play on the same day than those who were not immediately evaluated [15]. Determining the right time for athletes to return to play is of great importance, as concussed players are more prone to injury than other players [16].

The Aftermath

The aftermath of soccer-related concussions was also analyzed by a study exploring the relationship between specific sports and the subsequent occurrence of symptoms of common mental disorders (CMD) such as anxiety and depression. They found that former athletes who reported a history of four or five concussions were approximately 1.5 times more likely to report CMD symptoms [17]. Similarly, another study found that soccer is a common cause of post-concussion syndrome, which is characterized by a range of symptoms including headaches, insomnia, and irritability [18]. Yet, encouragingly, a longitudinal prospective study contributes substantial evidence indicating that concussions may not always lead to long-term abnormalities detectable through neuroimaging in terms of atrophy, focal signal abnormality, or other intracranial abnormalities [19].

It is crucial to acknowledge the limitations of this review, as the data primarily originates from high-income countries, with minimal representation from low-income countries, and no major neurologic symptoms were found specific to concussion. Additionally, the child population is inadequately considered in the majority of studies, showing an urgent need to expand the scope of research to enhance the generalizability of findings.

Soccer-related concussions are a cause of increasing concern among athletes of all ages. Comprehensive strategies should be developed to safeguard the well-being of soccer players, including well-established guidelines for in-field evaluation to ensure a timely diagnosis. Further research is needed to establish a clear relationship on the long-term effect concussions may have on soccer players.

## Conclusions

Literature has exposed critical insights into soccer research practices and findings. The data, derived from 45 articles meeting specified criteria, indicates a concerning gap in comprehensive concussion assessment, with only 42.2% of studies incorporating neurological evaluations, and a mere 15.5% utilizing the Sport Concussion Assessment Tool (SCAT) or similar. The inclusion of follow-up data in 35.5% of the studies underscores the need for more consistent long-term observation practices in soccer-related concussion research. A significant outcome is the increased vulnerability of midfielders to concussions resulting from player-player mechanisms, highlighting positional considerations in injury prevention. Certified athletic trainers appeared as primary evaluators, emphasizing their pivotal role in the initial assessment of concussions on the field.

The identified gaps, such as minimal low-income countries representation and child population, are inadequately considered in the majority of studies. This underscores the necessity for a more inclusive approach to soccer-related concussion research, ensuring representation from diverse socioeconomic backgrounds and age groups. This increase will not only contribute to a more comprehensive understanding of concussions but also facilitate the development of universally applicable protocols for diagnosis, management, and return-to-play strategies. Efforts should be directed toward addressing these limitations and promoting more inclusive and representative research practices in the study of soccer-related concussions.
